# Adherence to a Paleolithic Diet in Combination With Lifestyle Factors Reduces the Risk for the Presence of Non-Alcoholic Fatty Liver Disease: A Case-Control Study

**DOI:** 10.3389/fnut.2022.934845

**Published:** 2022-07-19

**Authors:** Mohammad Hassan Sohouli, Somaye Fatahi, Elma Izze da Silva Magalhães, Bianca Rodrigues de Oliveira, Pejman Rohani, Neda Ezoddin, Mehdi Mehdinezhad Roshan, Azita Hekmatdoost

**Affiliations:** ^1^Student Research Committee, Department of Clinical Nutrition and Dietetics, Faculty of Nutrition and Food Technology, Shahid Beheshti University of Medical Sciences, Tehran, Iran; ^2^Department of Nutrition, School of Public Health, Iran University of Medical Sciences, Tehran, Iran; ^3^Postgraduate Programme in Collective Health, Federal University of Maranhão, São Luís, Brazil; ^4^Pediatrics Gastroenterology, Department of Pediatrics, School of Medicine Children's Medical Center, Tehran University of Medical Sciences, Tehran, Iran; ^5^Department of Biology and Anatomical Sciences, School of Medicine, Shahid Beheshti University of Medical Sciences, Tehran, Iran; ^6^Department of Clinical Nutrition and Dietetics, Faculty of Nutrition and Food Technology, Shahid Beheshti University of Medical Sciences, Tehran, Iran

**Keywords:** NAFLD, Paleolithic, diet, lifestyle, chronic diseases, case-control

## Abstract

**Background:**

Evidence suggests the role of changing traditional lifestyle patterns, such as Paleolithic, to the modern lifestyle in the incidence and epidemic of chronic diseases. The purpose of this study was to investigate the associations between the Paleolithic diet (PD) and the Paleolithic-like lifestyle and the risk of non-alcoholic fatty liver disease (NAFLD) among an adult population.

**Materials and Methods:**

This case-control study was carried out among 206 patients with NAFLD and 306 healthy subjects aged >18 years. PD score was evaluated using a validated 168-item quantitative food frequency questionnaire. In addition, to calculate the Paleolithic-like lifestyle score, the components of physical activity, body mass index (BMI), and smoking status of the participants were combined with the score of the PD.

**Results:**

The mean PD and Paleolithic-like lifestyle scores were 38.11 ± 5.63 and 48.92 ± 6.45, respectively. After adjustment for potential confounders, higher scores of adherence to the PD diet conferred a protection for the presence of NAFLD [odds ratio (OR): 0.53; 95% confidence interval (CI): 0.28–0.98; *P* for trend = 0.021]. Furthermore, PD and healthy lifestyle habits were negatively associated with NAFLD (OR = 0.42, 95% CI 0.23–0.78; *P* for trend = 0.007).

**Conclusion:**

Our data suggest that the PD alone and in combination with lifestyle factors was associated with decreased risk of NAFLD in a significant manner in the overall population. However, prospective studies are needed to further investigate this association.

## Introduction

Non-alcoholic fatty liver disease (NAFLD) is characterized by excessive hepatic steatosis in the absence of other identifiable causes, such as excessive alcohol use and viral hepatitis (1). According to the systematic reviews and meta-analysis published in 2016, the estimated pooled prevalence of NAFLD worldwide is 25.2% (2), while in Iran, this prevalence is ~34% (3).

Non-alcoholic fatty liver disease is considered the most common cause of chronic liver disease worldwide, as well as it can affect extra-hepatic organs, increasing the risk for type 2 diabetes mellitus, cardiovascular diseases, and chronic kidney disease (2, 4). Moreover, patients with NAFLD have an increased risk of all-cause mortality (5), highlighting the need for efforts to prevent, treat, and/or retard the progression of the disease.

The pathophysiology underlying this disorder is complex and incompletely understood. A decade ago, proposed that a “multiple parallel hits hypothesis” in which insulin resistance, lipids, mitochondrial function, innate immunity, intestinal microbiota, genetic determinants, epigenetic mechanisms, environmental factors, cytokines, and lifestyle contribute to the evolution of inflammation and fibrosis in NAFLD (6–10) so that lifestyle interventions can be important for the management of patients with NAFLD (11). An international panel of experts proposes clear and simple criteria for a diagnosis of the fatty liver. The diagnosis is based on the recognition of underlying abnormalities in metabolic health with an acceptance that “MAFLD” instead of NAFLD may commonly co-exist with other conditions (12).

Modification in diet composition and eating patterns may be a sustainable approach to NAFLD prevention and treatment (13). A dietary pattern of growing interest is the Paleolithic diet (PD) pattern, also known as the Hunter-Gatherer or Paleo diet (14). This diet pattern, modeled after diets of people who lived as hunter-gathers during the Paleolithic Era, is characterized as a predominantly plant food-based diet, with a wide diversity of fruits, nuts, and vegetables, such as wild-plant foods that with high amounts of calcium and other minerals, includes lean meat, and is low in dairy, grains, sugar, and salt (15). Based on the nutrient profile, a PD may have potential benefits for the prevention and treatment of NAFLD (13). Nevertheless, there are relatively few studies that have examined its effects on NAFLD (13, 16).

The Paleolithic diet score was constructed as discordance between diet during the Paleolithic period and the present era (17, 18). However, considering the cumulative association between diet and lifestyle factors, studies have used this score alone or in combination with lifestyle factors (Paleolithic-like lifestyle score) in relation to the incidence of some chronic diseases and mortality, showing inverse associations (17–19). However, studies investigating the relationship between these scores in the NAFLD are still scarce.

Thus, the present study aimed to investigate the associations between PD scores alone and in combination with lifestyle factors and the risk of NAFLD among Iranian adults.

## Subjects and Methods

### Study Design and Population

The study protocol was ethically approved by the Regional Bioethics Committee of Shahid Beheshti University of Medical Sciences, Tehran, Iran (No: IR.SBMU.RETECH.REC.1400.802). The required sample size for the current work was calculated based on the hypothesis of 1.5 times decreased odds of NAFLD by PD in combination with lifestyle factors. Therefore, considering a type I error of 5%, the study power of 80%, and the ratio of controls to cases as ~1.5, we needed 206 cases and 306 controls for this project. In this case-control study, the newly diagnosed patients with NAFLD and healthy controls aged >18 years who attended the Hazrat Rasoul Hospital, Tehran, Iran between 2020 and 2021 were selected. NAFLD diagnosis (20–22) was based on the following criteria: chronic increment in liver enzymes (liver enzymes >19 U/L for women and >30 U/L for men), abstinence from alcohol intake, liver ultrasound compatible with NAFLD, having grades II and III NAFLD based on liver biopsy, and exclusion of other causes of liver disorders. Moreover, the case group was referred to our hospital for evaluation by FibroScan (20), with the FibroScan results showing a Controlled Attenuation Parameter (CAP) score of more than 237 and a fibrosis score of more than 7, and had the diagnosis of NAFLD confirmed by a gastroenterologist. Additionally, individuals without a history of NAFLD were recruited from the same hospital for the control group. The control group was selected from other outpatient clinics of our hospital, e.g., ophthalmology, otorhinolaryngology, and dermatology. The controls were required to have no history of chronic or inflammatory diseases (e.g., diabetes, cancer, gastrointestinal, or cardiovascular disorders, etc.) and to have a regular diet for the past 6 months. Two dietitians (Mh.S. and E.M.) monitored the sampling of the patients. The inclusion criteria for the control group were defined based on laboratory tests and the liver ultrasound (not suffering from any stages of hepatic steatosis). The individuals with long-term diet changes (due to a particular disease or weight loss), a history of renal and (or) hepatic disease (Wilson's disease, autoimmune liver disease, cirrhosis, non-alcoholic steatohepatitis (NASH), hemochromatosis, viral infections, or alcoholic fatty liver disease), cardiovascular disease, diabetic patients, malignancy, thyroid disorder, and autoimmune disease were not included in the study. At the beginning of the study, all the participants were asked to carefully answer the demographic, economic, and social questionnaire questions that evaluated age, employment status, education, smoking history, disease history status, use of specific medications (other than regular NAFLD drugs), and history of dieting in the past 6 months. The physical activity levels of the participants were estimated by the use of a validated short form of the International Physical Activity Questionnaire (Short IPAQ) (23). Data collection was conducted through interviews by a trained nutritionist.

### Dietary Assessment

The dietary intake over the previous year was obtained using a validated semi-quantitative food-frequency questionnaire (FFQ), which consisted of 168 food items (24). The FFQ consisted of a list of usual Iranian dietary items with standard serving sizes. For each food item, the average portion size consumed and the frequency of intake were obtained from self-reports on the FFQ. The frequency of intake for each food item included never, 2–3 times/month, 1 time/week, 2–4 times/week, 5–6 times/week, and daily. The portion sizes were reported in grams by using standard Iranian household measures (25). The daily nutrient consumption for each person were estimated by applying the United States Department of Agriculture's (USDA) National Nutrient Databank (26). The Nutritionist IV software (First Databank, San Bruno, CA, USA—modified for Iranian foods) was used to calculate the daily energy and nutrient intake of each participant.

### PD and Paleolithic-Like Lifestyle Scores Measurement

The study of Whalen et al. (17) was used to calculate the score of adherence to the PD. In summary, the food items obtained from each individual using the FFQ were divided into 14 food groups (such as vegetables, fruits, fruit and vegetable diversity score, lean meat, fish, nuts, and calcium as more PD characteristics and red and processed meat, dairy foods, sugar-sweetened beverages, baked goods, grains and starches, sodium, and alcohol as less characteristic PD). The fruit and vegetable diversity score in our study was defined as the number of components of the fruit and vegetable group consumed by each person. In order to score each food group, the intake of each food component was classified as quintiles (from 1 to 5, with “1” scores the minimum consumption in each food group and “5” scores the maximum) according to the distribution of consumption of the study population. PD scores ranged from 13 to 65 and higher scores indicated higher levels of adherence to the PD.

To calculate the Paleolithic-like lifestyle score according to the study of Cheng et al. (27), the components of physical activity, body mass index (BMI), and smoking status of the participants were combined with the score of the PD. Lifestyle factors, with the exception of smoking status, were classified according to the tertile distribution. For physical activity, a score of 5 was assigned to individuals with the highest tertile, and scores of 3 and 1 were assigned to middle and lower tertiles, respectively. In contrast, the scoring was reversed for BMI. In addition, for smoking status, scores of 5, 3, and 1 were assigned to non-smokers, ex-smokers, and smokers, respectively. Finally, all diet and lifestyle values for each participant were summarized to reflect adherence to the Paleolithic-like lifestyle score. The final score range in our study ranged from 16 to 80, and higher scores indicated better adherence.

Because all participants in the study were Muslim and did not consume alcohol, alcohol intake was not considered a component of these scores in our study.

### Anthropometric Measurement

An anthropometric evaluation was performed by the researchers. The patient's weight was measured with a Seca Portable Digital Scale made in Germany with an accuracy of 100 g with minimal coverage and without shoes. The patient's height needs were assessed with a Seca portable height gauge with an accuracy of 0.1 cm. In addition, the waist circumference (WC) in the middle area between the iliac crown and the last gear was determined with a Seca waist measuring device. The hip circumference was also measured in centimeters using the same measuring tape at its widest portion of the buttocks, with the tape parallel to the floor. BMI, after measuring weight and height with the mentioned method, was calculated using the formula weight (kg)/height^2^ (m). All anthropometric measurements were performed by the researcher to minimize observational variations.

### Biochemical Measurement

At the beginning and end of the study, after 10–12 h of fasting, 10 ml of venous blood was taken from the subjects by the laboratory technician. After clotting in the environment, the serum was isolated as soon as possible by centrifugation and kept at −70°C until sent to the laboratory for testing. Triglycerides (TG), high-density lipoprotein cholesterol (HDL-C), and fasting plasma glucose (FPG) concentration were measured using the Pars Azmoon Company Kit (ParsAzmun, Tehran, Iran) and enzymatic colorimetric method. Total cholesterol (TC) concentration was measured by enzyme photometry using the Pars Test Kit (ParsAzmun, Tehran, Iran). Low-density lipoprotein cholesterol (LDL-C) concentration was also calculated using the Friedewald formula (28): LDL-C = TC–TG/6–(HDL-C). Alanine aminotransferase (ALT) and aspartate aminotransferase (AST) were determined by commercially available enzymatic reagents (Pars Azmoon, Tehran, Iran) on auto analysis (BT-3000).

### Statistical Analysis

The Statistical Package Software for Social Science, version 21 (SPSS Inc., Chicago, IL, USA) was used for the statistical analysis. Kolmogorov-Smirnov's test and histogram charts were used to test the normality of the data. The baseline characteristics and dietary intakes were reported as mean ± standard deviation (SD) for quantitative variables, and number and percentages for qualitative variables. We compared the data between two groups using independent sample *t*-tests and chi-squared tests for continuous and categorical variables, respectively. The association of PD and Paleolithic-like lifestyle scores with the risk of NAFLD was assessed by applying logistic regression. The analyses were adjusted for probable confounders, e.g., sex and BMI, weight, WC, hip circumference, physical activity, smoking, education, and energy intake, fasting blood glucose (FBG), ALT, TG, HDL-C, and LDL-C. The odds ratio (OR) with a 95% confidence interval (CI) of NAFLD across quartiles of scores was calculated. *P*-values <0.05 were considered statistically significant.

## Results

The mean (±SD) age of the study population was 37.84 ± 8.70 years. The mean (±SD) BMI was 26.99 ± 4.32 kg/m^2^. The mean PD and Paleolithic-like lifestyle scores were 38.11 ± 5.63 and 48.92 ± 6.45, respectively.

The baseline characteristics of the study subjects are shown in Table 1. Compared with controls, NAFLD subjects had significantly higher BMI, weight, WC, hip circumference, ALT, FBG, TG, and LDL-C concentration, but had lower physical activity and HDL-C concentration. There was also a significant difference in the level of education between the case and control groups. However, no significant differences were found for other characteristics among cases and controls.

**Table 1:** Demographic, anthropometric, and lifestyle characteristics of participants in the case and control groups.

**Variables**	**Groups, mean (SD)**		***P*-value^**a**^**
	**With NAFLD** **(*n* = 206)**	**Without NAFLD** **(*n* =306)**	
Age, years	38.15 (8.43)	37.63 (8.88)	0.508
Female, *n* (%)	125 (60.7)	233 (76.1)	<0.001
BMI^b^, kg/m^2^	30.36 (3.77)	24.71 (2.97)	<0.001
Weight, kg	83.80 (10.12)	66.92 (8.87)	<0.001
Waist-circumference (cm)	102.34 (7.33)	85.17 (7.10)	<0.001
Hip-circumference (cm)	104.56 (8.36)	96.14 (5.77)	<0.001
Physical activity (Met.h/week)	1,114.57 (617.50)	1,625.59 (930.51)	<0.001
Smoking (yes), *n* (%)	16 (7.8)	8 (2.6)	0.007
**Drug use (other than regular NAFLD drugs) (yes)**, ***n*** **(%)**	9 (4.4)	7 (2.3)	0.184
Metformin	7 (3.4)	3 (1.0)	
Atorvastatin	2 (1.0)	4 (1.3)	
ALT (mg/dl)	58.50 ±24.1	20.53 ±13.01	<0.001
AST (mg/dl)	34.88 ±17.2	23.76 ± 9.6	0.17
FPG (mg/dl)	109.28 (39.39)	92.20 (36.22)	<0.001
TC (mg/dl)	184.79 (54.93)	182.84 (39.36)	0.728
TG (mg/dl)	180.39 (123.81)	130.98 (64.29)	<0.001
LDL-C (mg/dl)	121.16 (43.03)	109.13 (31.07)	0.007
HDL-C (mg/dl)	41.26 (16.71)	48.05 (10.87)	<0.001
Paleolithic diet (PD) score	41.4 (5.5)	44.2 (5.4)	<0.001
Paleolithic-like lifestyle scores	50.4 (5.5)	56.2 (5.9)	<0.001
**Education status**, ***n*** **(%)**
Lower than high school	28 (13.6)	37 (12.1)	<0.001
High school	81 (39.3)	112 (36.6)	
Higher than high school	97 (47.1)	166 (51.3)	

^a^*Obtained from ANOVA for continuous variables and chi-square for categorical variables*.

^b^*BMI: body mass index*.

*FPG, fasting plasma glucose; TG, triglyceride; TC, total cholesterol; HDL, high-density lipoprotein; LDL, low-density lipoprotein; ALT, alanine aminotransferase; AST, aspartate aminotransferase*.

Table 2 illustrates the macro- and micro-nutrients and the intakes of food groups in NAFLD patients vs. controls. NAFLD subjects had higher intakes of energy, carbohydrate, total fat, zinc, folate, total dairy, refined grains, and red and processed meats but lower intakes of total fiber, vitamin D, and vegetables as compared to controls. There were no significant differences between the NAFLD group and controls for all other dietary intakes.

**Table 2:** Dietary intakes of study participants across case and control groups.

	**Groups, mean (SD)**		***P*-value^**a**^**
	**With NAFLD (*n* = 206)**	**Without NAFLD (*n* = 306)**	
**Nutrients**			
Energy (Kcal/day)	2,360.71 (583.87)	2,212.76 (625.54)	0.007
Carbohydrate (g/day)	338.19 (96.99)	319.69 (102.10)	0.041
Protein (g/day)	79.09 (23.12)	74.96 (23.70)	0.052
Fat (g/day)	82.76 (29.23)	76.72 (27.95)	0.019
SFA (g/day)	27.52 (10.83)	26.18 (10.62)	0.166
Cholesterol (mg/day)	225.40 (116.10)	234.43 (155.78)	0.478
Fiber (g/day)	36.33 (18.99)	40.46 (23.42)	0.029
Sodium (mg/day)	4,669.27 (4,289.44)	4,281.67 (2,609.36)	0.205
Potassium (mg/day)	3,649.90 (1,211.97)	3,539.10 (1,318.38)	0.336
Iron (mg/day)	27.07 (13.03)	25.59 (3.85)	0.226
Calcium (mg/day)	1,251.37 (453.92)	1,181.10 (447.17)	0.084
Magnesium (mg/day)	378.58 (115.44)	360.72 (132.99)	0.117
Zinc (mg/day)	11.66 (3.49)	10.95 (3.60)	0.026
Vitamin C (mg/day*)*	136.69 (83.83)	131.45 (88.73)	0.503
Folate (mcg/day)	552.33 (170.33)	510.68 (153.00)	0.004
Vitamin E (mg/day)	11.86 (4.50)	11.15 (5.37)	0.116
Vitamin D (mcg/day)	1.71 (1.34)	2.05 (1.66)	0.014
Caffeine (mg/day*)*	132.81 (118.55)	132.27 (122.22)	0.961
**Food groups**
Total dairy (g/day)	481.04 (251.80)	421.68 (240.13)	0.007
Legume (g/day)	17.43 (26.09)	16.07 (18.86)	0.492
Nut (g/day)	6.60 (8.83)	6.47 (9.43)	0.873
Coffee (g/day)	17.71 (61.32)	15.63 (53.26)	0.684
Fish (g/day)	9.37 (8.96)	9.18 (7.83)	0.808
Whole grains (g/day)	91.50 (87.38)	99.54 (114.65)	0.394
Refined grains (g/day)	382.95 (219.80)	316.09 (153.74)	<0.001
Red and processed meat (g/day)	32.40 (25.12)	23.11 (19.58)	<0.001
Fruits (g/day)	355.72 (264.11)	353.06 (280.78)	0.914
Vegetables (g/day)	249.88 (129.32)	281.56 (142.78)	0.011

*SFA, saturated fatty acid*.

^a^*Obtained from ANOVA*.

General characteristics and dietary intake of subjects across the quartiles of PD scores are presented in Table 3. Compared with those in the lowest quartile of PD, subjects in the highest quartile had higher age but had lower weight and WC. No other significant difference was found in other general characteristics across quartiles of PD. In addition, individuals in the highest quartiles of PD had a higher intake of protein, saturated fatty acids (SFAs), cholesterol, potassium, iron, calcium, magnesium, zinc, vitamin C, total dairy, nut, fish, fruits, and vegetables, as well as a lower intake of sodium and refined grains.

**Table 3:** Socio-demographic characteristics, anthropometric variables, and dietary intake across the quartiles of Paleolithic diet (PD) score.

	**Quartiles of PD score**				***P*-value**
	**Q1**	**Q2**	**Q3**	**Q4**	
**Demographic variables**					
Age, years	36.56 (8.21)	36.92 (8.73)	37.37 (7.79)	40.51 (8.9)	0.001
Female, *n* (%)	88 (68.8)	85 (65.9)	94 (74.0)	91 (71.1)	0.537
BMI kg/m^2^	27.1 (4.17)	27.09 (4.38)	27.44 (4.34)	26.30 (4.35)	0.189
Weight, kg	75.05 (11.65)	73.60 (12.86)	75.99 (12.95)	70.23 (11.93)	0.001
Waist-circumference (cm)	93.98 (9.94)	92.41 (11.76)	92.47 (11.43)	89.45 (10.72)	0.010
Hip-circumference (cm)	100.25 (7.38)	99.46 (7.67)	100.06 (9.91)	98.36 (6.96)	0.235
Physical activity (Met.h/week)	1,479.32 (856.11)	1,448.03 (903.68)	1,345.53 (782.58)	1,454.54 (863.20)	0.608
Smoking (yes), *n* (%)	6 (4.7)	3 (2.3)	11 (8.7)	4 (3.1)	0.079
Disease history (yes), *n* (%)	6 (4.7)	1 (0.8)	1 (0.8)	8 (6.3)	0.055
Drug use (yes), *n* (%)	6 (4.7)	6 (4.7)	4 (3.1)	0 (0.0)	0.104
ALT (mg/dl)	34.17 (22.25)	36.58 (52.15)	36.63 (39.12)	26.09 (38.18)	0.316
AST (mg/dl)	29.13 (20.15)	28.75 (16.91)	25.61 (17.58)	24.12 (29.17)	0.119
FPG (mg/dl)	107.51 (55.52)	98.63 (34.93)	94.30 (28.20)	91.98 (28.22)	0.077
TC (mg/dl)	189.31 (42.10)	185.43 (47.86)	181.01 (45.46)	178.81 (44.58)	0.509
TG (mg/dl)	145.26 (65.48)	155.45 (82.95)	152.36 (130.93)	134.44 (62.33)	0.510
LDL-C (mg/dl)	120.74 (40.28)	112.86 (35.81)	108.83 (30.71)	110.16 (35.52)	0.194
HDL-C (mg/dl)	44.91 (11.94)	45.36 (13.03)	46.97 (12.27)	46.87 (12.27)	0.736
**Dietary intake**					
Energy (Kcal/day)	2,210.16 (621.36)	2,255.54 (600.48)	2,274.48 (650.04)	2,349.11 (576.52)	0.331
Carbohydrate (g/day)	320.72 (97.08)	323.09 (106.81)	325.04 (103.38)	339.70 (93.84)	0.426
Protein (g/day)	71.56 (23.68)	74.91 (22.21)	76.49 (24.26)	83.54 (22.55)	<0.001
Fat (g/day)	75.30 (28.46)	79.33 (26.90)	80.62 (31.05)	81.36 (27.80)	0.331
SFA (g/day)	24.18 (9.50)	26.72 (9.83)	27.21 (11.12)	28.77 (11.86)	0.007
Cholesterol (mg/day)	203.50 (104.48)	228.97 (190.54)	229.86 (110.26)	260.86 (137.41)	0.014
Fiber (g/day)	38.79 (26.66)	36.60 (19.74)	37.26 (18.25)	39.31 (18.15)	0.703
Sodium (mg/day)	5,174.65 (4,737.77)	4,356.74 (2,979.33)	4,353.93 (2,670.70)	3,865.12 (2,614.64)	0.019
Potassium (mg/day)	2,897.19 (1,004.50)	3,406.99 (1,155.69)	3,627.33 (1,143.36)	4,404.92 (1,310.24)	<0.001
Iron (mg/day)	24.25 (10.95)	24.85 (12.28)	25.82 (12.58)	29.84 (17.00)	0.004
Calcium (mg/day)	1,069.75 (434.33)	1,187.25 (441.69)	1,182.44 (419.21)	1,398.01 (448.60)	<0.001
Magnesium (mg/day)	334.66 (121.38)	357.93 (133.32)	371.52 (33.78)	407.65 (105.23)	<0.001
Zinc (mg/day)	10.56 (3.44)	11.16 (3.52)	11.14 (3.66)	12.08 (3.52)	0.007
Vitamin C (mg/day*)*	78.89 (44.81)	123.42 (90.13)	142.08 (64.99)	189.99 (97.43)	<0.001
Folate (mcg/day)	539.98 (178.94)	517.38 (159.72)	520.56 (153.93)	531.85 (152.19)	0.660
Vitamin E (mg/day)	10.96 (4.86)	11.31 (4.42)	11.64 (5.05)	11.84 (5.77)	0.533
Vitamin D (mcg/day)	1.64 (1.42)	1.76 (1.49)	1.85 (1.39)	2.40 (1.75)	<0.001
Caffeine (mg/day*)*	128.76 (125.16)	124.12 (101.01)	135.15 (113.79)	142.00 (139.94)	0.661
**Food groups**					
Total dairy (g/day)	395.26 (245.21)	442.37 (233.14)	422.51 (213.92)	521.96 (273.79)	<0.001
Legume (g/day)	17.23 (26.11)	14.90 (17.05)	15.48 (16.63)	18.85 (26.38)	0.471
Nut (g/day)	3.54 (5.06)	5.50 (5.29)	6.95 (9.95)	10.12 (12.81)	<0.001
Coffee (g/day)	10.22 (29.65)	18.78 (69.35)	17.77 (57.45)	19.09 (61.91)	0.549
Fish (g/day)	6.49 (6.39)	7.99 (6.22)	9.50 (7.81)	13.07 (10.61)	<0.001
Whole grains (g/day)	96.30 (106.01)	102.06 (129.65)	95.44 (100.36)	91.37 (75.68)	0.877
Refined grains (g/day)	429.21 (201.38)	346.40 (166.89)	326.55 (176.91)	269.65 (161.89)	<0.001
Red and processed meat (g/day)	30.12 (20.62)	27.81 (19.95)	25.78 (17.37)	23.66 (29.57)	0.119
Fruits (g/day)	176.57 (115.13)	324.89 (258.85)	384.00 (229.34)	531.52 (322.05)	<0.001
Vegetables (g/day)	183.68 (99.48)	243.22 (122.17)	284.96 (117.37)	363.74 (144.93)	<0.001

*Values are expressed as means [standard deviation (SD)] of 512 subjects*.

*P-values are resulted from the student's t-test*.

The ORs and 95% CIs for NAFLD subjects based on quartiles of PD and Paleolithic-like lifestyle scores are reported in Table 4.

**Table 4:** Odds ratio (OR) and 95% confidence interval (CI) for NAFLD based on Paleolithic diet (PD score) alone and in combination with lifestyle factors (combined score).

	**Quartiles of scores**				***P* for trend**
	**Q1**	**Q2**	**Q3**	**Q4**	
**PD score**					
Case/total	59/128	55/128	48/128	44/128	
Crude model	1.00 (Ref)	0.70 (0.42–1.15)	0.42 (0.25–0.70)	0.35 (0.20–0.59)	<0.001
Model 1*	1.00 (Ref)	0.74 (0.44–1.23)	0.59 (0.31–0.91)	0.56 (0.29–0.95)	0.018
Model 2^‡^	1.00 (Ref)	0.79 (0.46–1.35)	0.58 (0.31–0.94)	0.54 (0.29–0.98)	0.020
Model 3	1.00 (Ref)	0.80 (0.47–1.37)	0.55 (0.32–0.96)	0.53 (0.28–0.98)	0.021
**Combined score**					
Case/total	68/128	59/128	47/128	36/128	
Crude model	1.00 (Ref)	0.66 (0.38–1.14)	0.68 (0.39–1.19)	0.42 (0.24–0.73)	0.005
Model 1^†^	1.00 (Ref)	0.66 (0.38–1.15)	0.73 (0.41–1.28)	0.45 (0.25–0.79)	0.012
Model 2^¥^	1.00 (Ref)	0.77 (0.42–1.39)	0.69 (0.39–1.36)	0.44 (0.24–0.83)	0.006
Model 3	1.00 (Ref)	0.72 (0.39–1.33)	0.65 (0.35–1.20)	0.42 (0.23–0.78)	0.007

*Binary logistic regression was used to obtain OR and 95% CI*.

*Model 1: adjusted for sex and BMI.

^†^*Model 1: adjusted for sex*.

^‡^*Model 2: Model 1 + weight, hip circumference, physical activity, smoking, education, and energy intake*.

^¥^*Model 2: Model 1 + weight, hip circumference, education, and energy intake*.

*Model 3: Model 2 + fasting plasma glucose, ALT, triglyceride, HDL-C, and LDL-C*.

In crude and first adjusted model (based on age and BMI), there was a significant association for PD score in the highest quartile when compared with the lowest quartile (OR = 0.35, 95% CI 0.20–0.59; *P* for trend < 0.001; OR = 0.56, 95% CI 0.29–0.95; *P* for trend = 0.018, respectively). Furthermore, after adjusting for confounders according to the final model, higher scores of adherence to the PD conferred a protection for the presence of NAFLD (OR: 0.53; 95% CI: 0.28–0.98; *P* for trend = 0.021). There was a significant relationship between reduced odds of NAFLD for those subjects with the highest score of Paleolithic-like lifestyle, when compared to subjects with the lowest score, both in the crude (OR = 0.42, 95% CI 0.24–0.73; *P* for trend = 0.005) and the final adjustment (OR = 0.42, 95% CI 0.23–0.78; *P* for trend = 0.007) models.

## Discussion

In the present study, the consumption of PD was associated with a lower chance of occurrence of NAFLD. Furthermore, the Paleolithic-type lifestyle, characterized by the combination of the PD and healthy lifestyle habits, was also negatively associated with NAFLD.

Our results suggest that adherence to the PD can protect the development of NAFLD. Recently, Fraczek et al. (29) in a meta-analysis study of randomized clinical trials evidenced the health benefits of adopting the PD. This meta-analysis found that even with short-term consumption, the PD contributed to normalizing blood pressure, improving lipid profile, with a reduction in total cholesterol (TC), TG, and LDL-C and increased HDL-C, sensitivity to insulin and glucose tolerance, and decreased body weight, fat mass, and WC. These results were also confirmed by Shah et al. (19) in a French prospective study, in which the PD and adherence to a Paleolithic lifestyle proved to be favorable alternatives for the prevention of type 2 diabetes and hypertension. Furthermore, it has been suggested that adherence to the PD was associated with a lower chance of systemic inflammation and oxidative stress (30). Considering the evidence presented and the results found in the present study, the PD can be an effective strategy for the prevention of NAFLD.

The potential benefits of PD on health may be due to a food composition that is marked by high consumption of fruits, vegetables, fish, and nuts, as well as limiting the consumption of processed and ultra-processed foods, added sugar, salt, and vegetable oils (31). As a result, a diet rich in antioxidants, fibers, monounsaturated and polyunsaturated fatty acids, potassium (32), vitamins B, D, E, and K, coenzyme Q10, alpha lipoic acid, and polyphenol acid (29).

Evidence suggests that micronutrients, such as vitamins A, C, D, and E, carotenoids, zinc, copper, iron, selenium, and magnesium, may have beneficial effects on NAFLD, due to its antioxidant, antifibrotic, immunomodulatory, and lipoprotective properties (33).

The findings of the present study are consistent with recent studies that have highlighted the protective role of healthy eating against NAFLD. In a case-control study conducted with Iranian adults, it was observed that the consumption of a dietary pattern characterized by the consumption of vegetables, legumes, fruits, and dairy products with low-fat content was associated with lower chances of occurrence of NAFLD (34). Another case-control study with Iranian adults (35) found that NAFLD was inversely associated with a healthy dietary pattern, which consisted of relatively high consumption of fish, skinless poultry, low-fat dairy products, fruits, vegetables, nuts, oil, and garlic. In this study, the authors found that individuals in the highest tertile of the healthy eating pattern scores had lower odds of NAFLD than those in the lowest tertile, even after adjustment for potential confounding factors (OR: 0.30; 95% CI: 0.13–0.68).

It is already established in the literature that unhealthy eating, characterized by a diet rich in calories, sugars, saturated fats, low in polyunsaturated fatty acids, fiber, and micronutrients, is a determining factor for both the development and progression of NAFLD (36). Recently, a review article highlighted that individuals with NAFLD share a common dietary pattern, identified by low consumption of whole grains, cereals, fruits, and vegetables and high consumption of red meat, offal, refined grains, and sugars (37). In addition, evidence from a systematic review and meta-analysis demonstrated that Western dietary patterns containing high consumption of processed foods, red meats, high-fat dairy products, and refined grains can significantly increase the occurrence of NAFLD (OR: 1.56; 95% CI: 1.27–1.92). In addition, pieces of evidence showed that cooking meat at high temperatures for a long duration forms heterocyclic amines (HCAs), which are related to oxidative stress and NAFLD (38). In a systematic review study (39), in line with our results, it has been stated that the PD has beneficial effects on various risk factors related to NAFLD in various ways. So PD modulates hyperglycemic carbohydrates and, on the other hand, by eliminating insulin-tropic dairy, regulates insulin/insulin-like growth factor 1(IGF-1) signaling, which has recently been recognized as one of the risk factors associated with NAFLD (39, 40). The study also found that PD improved insulin resistance and dyslipidemia, thereby preventing the development of NAFLD (39). This diet also has a lower fructose content than other common diets, which can also prevent the development of NAFLD (39).

It is important to note that the Paleolithic lifestyle score also played a protective role against NAFLD in our study. This demonstrates that in addition to diet, each of the healthy lifestyle factors, which included physical activity, adequate BMI, and not smoking, played an important role in preventing NAFLD. It has been shown that NAFLD is affected by obesity, sedentary lifestyle, and smoking, in addition to other individual factors and environments (41).

Regarding anthropometric and metabolic markers, BMI, WC, FPG, TG, and LDL-C were significantly higher in the NAFLD group as compared to the control group, while HDL-C levels were significantly lower in cases as compared to controls. Similar results were observed in other publications (42, 43). This finding is expected since NAFLD is closely linked to obesity, insulin resistance, and dyslipidemia (44), as well as being considered the hepatic manifestation of the metabolic syndrome (45).

This study has strengths and limitations. Among the limitations, the study design makes it impossible to assess the causal relationship. Another limitation is that despite the adjustment for several possible confounding factors, it is not possible to exclude the possibility of the existence of some potential confounding factor that has not been included in the analyses. In addition, self-reported information about food consumption can cause recall bias.

The current research has several strengths. As far as we know, this is the first study to assess the association between consumption of the PD alone and in combination with lifestyle factors and the risk of NAFLD among Iranian adults, in which trained personnel was employed to interview and collect food frequency questionnaires. Our sample size was sufficient and we tried to eliminate the impact of confounders by adjusting for a wide range of variables and by using a validated questionnaire. In addition, we could not examine the consumption of alcohol due to the fact that the participants were Muslims can be another limitation of this study. However, because both the control group and the study case group did not consume alcohol, a review of this component of the PD could not affect the results.

In conclusion, by calculating the study power by almost 80%, the present study provides pieces of evidence that PD and adherence to a healthy lifestyle were associated with decreased risk of NAFLD. Our results support previous findings of the protective role of healthy eating and living habits for NAFLD. Furthermore, they can serve as strategies for preventing and even controlling the progression of NAFLD.

## Data Availability Statement

The raw data supporting the conclusions of this article will be made available by the authors, without undue reservation.

## Ethics Statement

This study was approved by the research council and Ethics Committee Iran University of Medical Sciences, Tehran, Iran. The patients/participants provided their written informed consent to participate in this study.

## Author Contributions

AH and MS contributed in conception, design, statistical analysis, and supervised the study. MS, BO, EM, and SF contributed to data collection and manuscript drafting. All authors contributed to the article and approved the submitted version.

## Conflict of Interest

The authors declare that the research was conducted in the absence of any commercial or financial relationships that could be construed as a potential conflict of interest.

## Publisher's Note

All claims expressed in this article are solely those of the authors and do not necessarily represent those of their affiliated organizations, or those of the publisher, the editors and the reviewers. Any product that may be evaluated in this article, or claim that may be made by its manufacturer, is not guaranteed or endorsed by the publisher.
